# Isolation and characterization of bacteriophages from clinical enterohemorrhagic *Escherichia coli* strains

**DOI:** 10.1128/spectrum.00597-25

**Published:** 2025-09-05

**Authors:** Jessie Vandierendonck, Adam Valcek, Van Son Nguyen, Didier Vertommen, Surbhi Malhotra-Kumar, Henri De Greve, Remy Loris

**Affiliations:** 1 VUB-VIB Center for Structural Biology, Vlaams Instituut voor Biotechnologie and Structural Biology Brussels, Vrije Universiteit Brussel, Brussels, Belgium; 2 UCLouvain, de Duve Institute, Proteomics Platform (MASSPROT), Brussels, Belgium; 3 Laboratory of Medical Microbiology, Vaccine and Infectious Diseases Institute, University of Antwerp 198686, , Antwerp, Belgium; Centre de Biologie Integrative, Toulouse, France

**Keywords:** temperate bacteriophages, enterohemorrhagic *E. coli*, Shiga toxin, bacteriophage receptor, genome sequencing

## Abstract

Temperate bacteriophages play a pivotal role in the biology of their bacterial host. Of particular interest are bacteriophages infecting enterohemorrhagic *E. coli* (EHEC) due to their significant contribution to the pathogenicity of its host, most notably by encoding the key virulence factor of this pathogen, the Shiga toxin. To better understand the role of EHEC phages on the functionality of its host, we isolated eight temperate phages from clinical EHEC isolates and characterized their genomic composition, morphology, and receptor targeting. Morphological analysis identified one long-tailed siphophage, targeting the OmpC receptor for host recognition, whereas the other seven phages are short-tailed podophages and target the essential BamA protein. Genomic characterization revealed significant variations between the long- and short-tailed phages. Five of the eight isolated phages encode the potent Shiga toxin. Comparative analysis displays the typical lambdoid mosaicism, indicative of horizontal gene transfer driving evolution. These findings provide insights into the genetic and morphologic diversity and receptor specificity of EHEC phages, highlighting their role in the evolution and pathogenicity of clinical EHEC strains.

**IMPORTANCE:**

Characterizing bacteriophages from clinical EHEC isolates is crucial in understanding the mechanisms underlying bacterial evolution and virulence. Despite the clinical relevance of EHEC bacteriophages, they remain underexplored, and particularly phage receptors are often not characterized. Studying temperate EHEC phages is essential in the development of strategies to address the global burden of these foodborne infections. Notably, identifying the phage receptors is critical in unraveling the specific interaction between phage and host. Knowledge of the phage receptors can provide insights into the mechanisms of phage infection, host range, and bacterial resistance and is fundamental in the design of targeted therapies like new antimicrobials, phage therapy, or prevention of those infections.

## INTRODUCTION

Shiga toxins are encoded by temperate bacteriophages and are the primary virulence factor of Shiga toxin-producing *Escherichia coli* (STEC). Among these STEC strains, Shiga toxin-producing enterohemorrhagic *E. coli* (EHEC) is a significant subset responsible for foodborne infections that can lead to severe gastrointestinal illness (1). These infections result in bloody diarrhea due to extensive damage to the intestinal epithelium by forming attaching and effacing (A/E) lesions and, in severe cases (5%–15%), may progress into the life-threatening hemolytic uremic syndrome (HUS) (2). Since EHEC was first identified after an outbreak in 1982 (3), numerous outbreaks have been documented globally (4). A recent outbreak in October 2024 in the United States has regained awareness for this pathogen (5). Treatment of these infections with certain antibiotics is complicated due to the triggering of the SOS response in EHEC, which results in the increased production of the Shiga toxin, leading to a higher risk of developing HUS (6). The role of temperate phages, which integrate their DNA as prophages into bacterial genomes, is critical in understanding the biology, evolution, and virulence of their lysogens. The introduction of the phage’s genetic material can profoundly impact host biology, including the acquisition of virulence factors such as the Shiga toxin (7). Upon exposure to environmental stressors, the lytic cycle of these prophages can be induced. This results in the production and release of not only new phage progeny but also of the Shiga toxin, contributing to pathogenesis (8).

Shiga toxin-encoding phages (Stx phages) are lambdoid phages sharing a functional genetic organization and lifestyle similar to that of phage Lambda. These phages are capable of recombining with each other, which can result in the formation of hybrid phages with functional and potentially novel properties (9). This modular structure or mosaic pattern contributes to the diversity and heterogeneity of Stx phages (10). Understanding the dynamics of Stx phages is essential for elucidating their contribution to bacterial virulence and for developing strategies to mitigate the public health threat posed by EHEC and related pathogens.

Despite their abundance and biological relevance, relatively few phages have been thoroughly characterized (11, 12). Here, we characterize eight temperate phages that were isolated from EHEC clinical isolates. The characterization covers their genomic organization, morphology, and host receptors.

## MATERIALS AND METHODS

### Bacterial strains

Clinical EHEC were isolated between 1987 and 1996 from fecal samples collected from patients in the laboratory of Microbiology of the University hospital at Vrije Universiteit Brussel (UZ, Brussel) (13–15). They were grown in low salt O157 medium (10 mM CaCl_2_, 10 g/L tryptone, 5 g/L yeast extract, 50 mM Tris-HCl, pH 7.5) or Mueller Hinton (MH) medium (2 g/L beef infusin solids, 1.5 g/L starch, 17.5 g/L casein hydrolysate, pH around 7.4) for the isolation of bacteriophages. *E. coli* K514 (16) was further used for propagation of the isolated bacteriophages.

### Bacteriophage isolation

Each bacterial EHEC strain was grown overnight by incubation at 37°C, 170 rpm, in low salt O157 medium or MH medium. Bacteriophages were isolated after spontaneous prophage induction overnight: 1 mL of the overnight culture was centrifuged at 10,000 rpm for 2 min. Bacteriophages present in the supernatant were then filtered through a 0.22 µm Millipore filter. To check for the presence of phages, the filtrate was spotted on *E. coli* K514 as the host strain using the standard agar overlay plaque assay. For this, 0.1 mL of overnight-grown *E. coli* K514 was mixed with 4 mL of O157 soft agar (0.7% agar) and poured onto O157 bottom agar medium (1.5% agar). After the top agar was solidified, 10 µL droplets of the 10-fold serial-diluted, filtered supernatants were applied on top and incubated overnight at 37°C. The next day, the result of spontaneous induction was evaluated by observing plaque formation.

### Bacteriophage enrichment

The isolated bacteriophages were purified by stabbing a plaque using a sterile tip or a Pasteur pipette to make sure that one single plaque was selected. Mild suction was applied to draw the plaque into the pipette. Then, the tip was placed into 1 mL of O157/MH medium supplemented with a droplet of chloroform to diffuse the phage particles. To enrich the bacteriophages, phages corresponding to 10^5^ plaque-forming units were mixed with 0.1 mL of plating *E. coli* K514 and incubated at 37°C for 20 min. Afterward, 4 mL of melted 0.7% top agar was added and poured onto a plate containing O157/MH bottom agar. This plate was incubated overnight at 37°C. The following day, when nearly confluent lysis was achieved, the soft agar was gently scraped off with a sterile, L-shaped spreader into a sterile centrifuge tube. Optionally, 1–3 mL of the liquid O157/MH medium was added to the plate to rinse off any remaining soft agar. Then, 0.1 mL chloroform was added and mixed by shaking for 15 min at 37°C. After centrifugation at 7,000 × *g* for 10 min at 4°C, the supernatant was recovered. Chloroform was added to a final concentration of 0.3%, and the obtained phage stock was stored at 4°C. The titer of the phage stock was determined using a standard agar overlay plaque assay. A serial 10-fold dilution of the phage stock was prepared in liquid O157/MH medium, and 10 µL of each dilution was spotted onto a bacterial layer of K514. The next day, the number of plaques was counted in the appropriate dilution, and the titer was determined. Finally, the purified phages were kept at −80°C as glycerol stocks (final concentration of 20% glycerol) and integrated as prophages in K514 lysogens (final concentration of 40% glycerol).

### Genome sequencing

For the isolation of bacteriophage DNA, either the classic phenol-chloroform extraction method (phi330 and phi345) or the Genomic Mini AX Phage kit (phi296, phi315, phi346, phi349, phi367, and phi419) (A&A Biotechnology, Poland) was employed. This latter kit was used for the phage samples subjected to nanopore sequencing to avoid potential interference from residual phenol. DNA concentrations and purity were assessed using a Qubit 4 Fluorometer (Invitrogen) and a NanoDrop One spectrophotometer (Isogen), respectively. The genome of phi330 was Sanger-sequenced and manually assembled as follows. Phi330 DNA was separately digested with restriction enzymes EcoRI, MunI, and EcoRV. The EcoRI and MunI fragments were cloned into dephosphorylated EcoRI-digested pUC8, and the EcoRV fragments into dephosphorylated SmaI-digested pUC8. After transformation in CaCl_2_-competent DH5α cells, white colonies were selected on LB medium supplemented with ampicillin (100 µg/mL final concentration), isopropyl-β-D-thiogalactopyranoside (final concentration 1 mM), and 5-bromo-4-chloro-3-indolyl-β-D-galactopyranoside (final concentration 100 µg/mL). White colonies were picked and sequenced manually with vector primers 5′-agcggataacaatttcacacagga and 5′-cgccagggttttcccagtcacgac. If needed, new sequencing primers were designed to achieve the complete sequence of the cloned fragments. After sequencing and comparing the subclone sequences, the complete genome of phi330 was assembled. The genomic DNA of phi345 was paired-end sequenced using Illumina technology (2 × 251 bp). Raw reads in .fastq format underwent quality filtering (Q-score ≥ 20) and adapter trimming using Trimmomatic (17), followed by *de novo* assembly with SPAdes v. 3.15.2 using the --careful option (18). Assembled genomes were subjected to coverage (>8×) and length (>500 bp) filtering. Additionally, the genomes of phi296, phi315, phi346, phi349, phi367, and phi419 were sequenced using nanopore technology at the VIB Nucleomics Core (https://nucleomicscore.sites.vib.be/en). A multiplex library of the various phages was prepared with the ONT rapid barcoding gDNA library kit. Sequencing was conducted on a Flongle flow cell, with base calling performed on a GridION platform using Guppy 7.1.4 in “Super-accurate base-calling, 400 bp” mode. The complete phage genomes were annotated using Pharokka (19). Additional PCR amplification and Sanger sequencing were performed for phages phi296, phi315, and phi367 to complete the final assembly. PCR primer pairs were designed at the borders of the obtained contigs. For phi296, primers S87 (5′-tgctatgaccaagctgccgt) and S88 (5′-gacaggaagcgatcacgtag) were used; for phi315, primer pairs S72-S73 (5′-gcacaacattataggtgac; 5′-ctgatgtttgcggctttcg), S74-S75 (5′-tatggtgcatggatgcctga; 5′-gaaagaacggcgttcattgac), S70-S71 (5′-atgcgcaggaaccaaccatg; 5′-gtatgtcttccaaacgtagc), and S76-S77 (5′-tcattcttccggaatgtcat; 5′- cggaacacggaactcaatta) were used; and for phi367, S85-S86 (5′-tgactatgccaaattgcagg; 5′-gtgacgcgaaaagatgcctg), S70-S99 (5′-atgcgcaggaaccaaccatg; 5′-gtgccaatcaatggaaactg), S95-S96 (5′-cgtacgccggatgagaatca; 5′-cgttctggtccggtacggtc), and S91-S92 (5′-tagctcaatgtacgaggaag; 5′-cagtaactgcggggctttaag) were used.

### Morphology analysis

Bacteriophages were purified using a continuous cesium chloride gradient with an initial density of 1.5 g/L before undergoing ultracentrifugation. For this process, 7.5 g of CsCl was added to a 10 mL phage stock in an open-top 13.2 mL ultracentrifuge tube (Beckman). The mixture was centrifuged for over 24 h at 4°C and 36,000 rpm in a Beckman SW 41 Ti rotor ultracentrifuge. Phage bands were isolated by carefully removing the layer above the phage-containing band and collecting the white phage-containing band using a pipette. The isolated phages were then dialyzed to remove CsCl using a buffer containing 100 mM CaCl_2_, 10 mM MgSO_4_, and 50 mM Tris (pH 8). Subsequent analysis was conducted via transmission electron microscopy (TEM) at the VIB-VUB Facility for Bio Electron Cryogenic Microscopy (BECM) in Brussels, Belgium. A 3 µL drop of the bacteriophage sample was placed on a 300 mesh EM grid for 30 s. The grids were then negatively stained with 20 µL of 2% uranyl acetate and allowed to dry for 30 min before observation. The samples were examined using a JEOL 1400 microscope at a magnification of ×50,000.

### Mass spectrometry

The dialyzed CsCl-purified phages were also used for mass spectrometry analysis at the MASSPROT core facility of the Duve Institute. First, acetone precipitation was performed on the samples, followed by denaturation in 4 M urea. To prepare the proteins for mass spectrometry analysis, reduction and alkylation of cysteine bridges were carried out. Subsequently, a cleanup step was conducted using solid-phase extraction (SPE) on a C18 column before overnight digestion with trypsin. For the liquid chromatography-tandem mass spectrometry (LC-MS/MS) analysis, a nano-LC was performed on a C18 Easyspray column (0.75 µm × 25 cm, Thermo Scientific). Data-dependent acquisition (DDA) was carried out on an Orbitrap Fusion Lumos mass spectrometer, with a gradient time of 90 min. For protein identification, Proteome Discoverer 2.5 software was used, employing both Sequest and MSAmanda as search engines. The search was conducted against a phage protein database that was composed of the ORFs, resulting from the previously obtained phage sequences. The results were filtered to ensure a false discovery rate (FDR) of less than 5%.

### Transgene insertion using Tn7

The *ompC* gene was inserted in the chromosome of *E. coli* strain BW25113 Δ*ompC768::kan*, under control of the IPTG-inducible *lac* promoter (P*lac*) via Tn7 transposon (20). First, P*lac-ompC* was synthesized at Twist Bioscience, flanked by 25 bp of overhang with the XhoI/PacI-digested pGRG37 plasmid. This construct was then cloned into the XhoI/PacI-digested pGRG37 plasmid using the NEBuilder HiFi DNA Assembly Master Mix. The cloned plasmid was transformed into CaCl_2_-competent *E. coli* BW25113 Δ*ompC768::kan* cells and selected on LB ampicillin (100 µg/mL) plates at 32°C overnight (as this plasmid is temperature-sensitive). A few colonies were purified on LB ampicillin plates and grown again at 32°C overnight. The next day, precultures were grown overnight in liquid LB medium at 32°C while shaking. The cultures were diluted and plated on LB to obtain single colonies and grown overnight at 42°C to allow loss of the heat-sensitive plasmid. Colonies were purified at 42°C on LB plates and LB plates supplemented with ampicillin. PCR was done on ampicillin-sensitive colonies using primers 5′-gatgctggtggcgaagctgt and 5′-gatgacggtttgtcacatgga. These primers flank the Tn7 attachment site next to the *glmS* gene (21). PCR on BW25113 Δ*ompC768::kan* will yield a product of 678 bp. When the P*lac-ompC* gene is inserted into the attachment site, a PCR product of 2260 bp will be observed.

### BamA-his production and purification

The *E. coli* K12 recombinant BamA expression construct consists of the mature *E. coli* BamA sequence with the N-terminal OmpA signal sequence followed by a Strep II tag, a 6× His tag, and a TEV-cleavage site (22). This construct was then transformed into *E. coli* BL21AI, and the recombinant his-tagged BamA (BamA-his) was purified following the protocol (22) with some modifications. Briefly, 4 L of BamA-his expressing cells was grown in LB medium at 37°C until OD 0.6–0.8, then induced by 200  µg/L anhydrotetracycline at 30°C for overnight incubation, and harvested by centrifugation at 5,000 × *g* for 10 min. Wet cells, 20 g, were then resuspended in 200 mL of the total cell lysis buffer containing 50  mM Tris, pH 8, 300  mM NaCl, 10  mM imidazole, 100  µg/mL lysozyme, 50  µg/mL DNase I  +  5  mM Mg^2+^, 0.4  mM AEBSF, 1  µg/mL leupeptin, and 1% β-d-dodecyl maltoside (DDM). After 2 h incubation at 4°C, the lysate was classified using an ultracentrifuge with a Ti 45 fixed-angle rotor at 40,000 rpm, 4°C, and 45 min, before being loaded onto a pre-equilibrated HisTrap FF Ni-IMAC column (Cytiva). Contaminants were washed with 12 column volumes (CV) of buffer A (50  mM Tris, pH 8, 300  mM NaCl, 10 mM Imidazole, and 0.03% DDM), and then, 12 CVs of 90% buffer A with 10% buffer B (50  mM Tris. pH 8, 300  mM NaCl, 500 mM Imidazole, and 0.03% DDM). The column was further washed with 2 CVs of buffer A without Imidazole but supplemented with 0.1% SDS, to remove BAM components, followed by 2 CVs of buffer A before elution. BamA was eluted using a mixture of 40% buffer A and 60% buffer B, concentrate,d and loaded onto a Superose 6 10/30 column (GE Healthcare), pre-equilibrated with 20  mM Tris, pH 8, 150  mM NaCl, and 0.03% DDM. BamA-containing fractions, by SDS-PAGE, were pooled and stored at −80°C.

### BamA-his competition assay

Phage stocks had a titer of around 10^8^–10^10^ PFU/mL. From these phage stocks, 10 µL (around 10^6^–10^8^ phages) was mixed with 10 µL of purified BamA-his purified protein (2 mg/mL), corresponding to 1.36 × 10^14^ molecules, a protein excess of at least 1 million. As a negative control, 10 µL of phages was mixed with 10 µL of gel filtration buffer (20 mM Tris, pH 8, 150 mM NaCl, and 0.02% DDM). These mixtures were added to an Eppendorf tube with 10 µL of Nickel beads (binding capacity of ≥40 mg/mL), which will trap the phage-bound BamA-his protein. After short centrifugation, 10-fold serial dilutions were made of the supernatant up to 10^−6^ in gel filtration buffer and spotted on *E. coli* K514 bacteria.

## RESULTS

### Phage isolation

EHEC phages play a critical role in shaping the pathogenicity of their bacterial host and are therefore relevant to study. We had 28 clinical enterohemorrhagic *E. coli* (EHEC) isolates (13) and decided to use these isolates in a search for novel temperate EHEC phages. For eight of the 13 initially isolated phages, a sufficiently high titer could be obtained (> 10^9^ PFU/mL) that stayed stable for several weeks when kept at 4°C (Table S1). These phages were further characterized by determining phage morphology using TEM, sequencing their genomes, performing mass spectrometry, and identifying the phage receptor.

### Morphology

**Fig 1:**
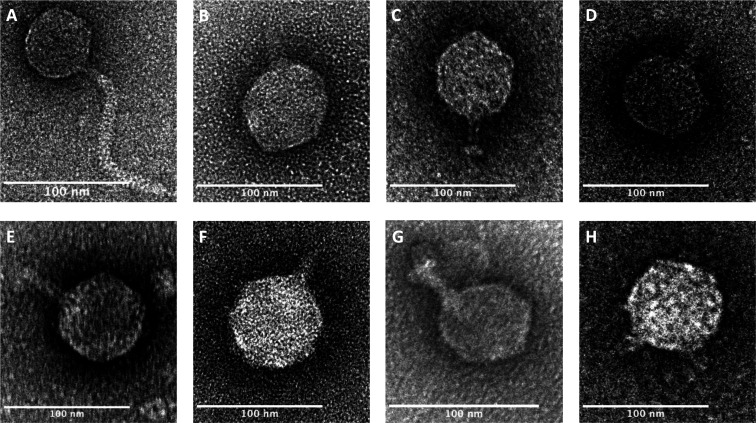
Negative staining electron microscopy images of the different phages. (**A**) phi330; (**B**) phi296; (**C**) phi315; (**D**) phi345; (**E**) phi346; (**F**) phi349; (**G**) phi367; (**H**) phi419; scale bar 100 nm.

Phage morphology was determined using negative-stain transmission electron microscopy. Based on their tail structures, the phages can be classified into two distinct morphological groups. All phages exhibit an icosahedral head morphology. However, phage phi330 displays a unique tail structure compared with the other phages (Fig. 1). Phage phi330 has an icosahedral head of approximately 55 nm in diameter with a long, flexible tail measuring about 145 nm, placing it within the group of siphophages (Fig. 1A). In contrast, all other phages possess an icosahedral head that ranges from 57 nm (phi367) to 72 nm (phi419) in diameter with a short tail of roughly 30 nm (Fig. 1B through H), suggesting that they belong to the group of podophages (23).

### Phage taxonomy

In addition to morphology-based classification, the phages were taxonomically classified using the binomial nomenclature from the International Committee on Taxonomy of Viruses (ICTV) (24). All tailed dsDNA phages fall within the class *Caudoviricetes*. Nucleotide BLAST analysis (25) using default settings and excluding “*Escherichia coli* (taxid 562)” was performed against databases for “phages with short tails” (short-tailed phages) and “phages with long, non-contractile tails” (phi330). This further refined classification: phages with over 70% genome identity were assigned to the same genus, whereas those with over 95% identity were designated as the same species. As listed in Table 1, phages were clustered using VIRIDIC analysis based on their intergenomic similarities (26). We identified three different genus clusters and seven species clusters. Phages phi346 and phi349 share more than 95% sequence identity with each other and Oslovirus PA2, placing them within the same species cluster. Similarly, phi345 and phi296 exhibit over 95% genome identity with Traversvirus P27 and Traversvirus tv86, respectively. Although phi346, phi349, and phi367 are classified within the same genus cluster of Osloviruses, phi345, phi296, phi315, and phi419 are categorized as Traversvirus, yet different from the distinct phi330 that is a Pankowvirus. The phages were named based on the naming criteria proposed by Kropinski et al. (27).

**TABLE 1:** Morphological classification, VIRIDIC clustering analysis, and taxonomy of the eight isolated phages in this study

Phage	Morphological group	Genus cluster	Species cluster	Genus-species	Virus name
phi330	Siphovirus	2	3	Pankowvirus	vB_EcoS_phi330
phi346	Podovirus	3	5	Oslovirus PA2	vB_EcoP_phi346
phi349	Podovirus	3	5	Oslovirus PA2	vB_EcoP_phi349
phi367	Podovirus	1	6	Oslovirus	vB_EcoP_phi367
phi345	Podovirus	1	4	Traversvirus P27	vB_EcoP_phi345
phi296	Podovirus	1	1	Traversvirus tv86	vB_EcoP_phi296
phi315	Podovirus	1	2	Traversvirus	vB_EcoP_phi315
phi419	Podovirus	1	7	Traversvirus	vB_EcoP_phi419

### Genome organization

The complete genomes of the eight phages were sequenced using Sanger sequencing for phi330, short-read Illumina technology for phi345, and long-read Nanopore technology for phi296, phi315, phi346, phi349, phi367, and phi419. For long-read nanopore sequencing, multiple potential assemblies were obtained for phages phi296, phi315, and phi367. To get the final and correct assembly, additional Sanger sequencing of overlapping PCR fragments of the unresolved contigs was done. Further information on the assembly for phages phi296, phi315, and phi367 is present in the supplemental data as sequence alignment maps (Assembly phi296.bam, Assembly phi315.bam, and Assembly phi367.bam).

Each phage genome consists of double-stranded DNA (dsDNA) with an approximate size of 60 kb and a GC content of around 50% (Table 2). After annotation of the phage genomes, three tRNA genes and approximately 100 coding DNA sequences (CDSs) were identified per genome. The majority of the CDSs (around 60) encode hypothetical proteins with unknown functions; the remaining CDSs encode known phage proteins.

**TABLE 2:** Genome characteristics of the eight isolated phages in this study

Parameter	phi330	phi296	phi315	phi345	phi346	phi349	phi367	phi419
Size (bp)	55.911	57.221	59.532	62.385	63.554	62.509	58.966	60.662
GC %	51%	50%	49%	49%	50%	50%	50%	49%
CDSs	124	98	101	110	104	98	109	102
Hypothetical proteins	55	56	57	44	60	55	67	56
Upper strand	81	62	63	75	65	59	70	70
Lower strand	43	36	38	39	39	39	39	32
Accession number	PV189401	PV189400	PV189403	PV189402	PV189404	PV189405	PV189406	PV189407

**Fig 2:**
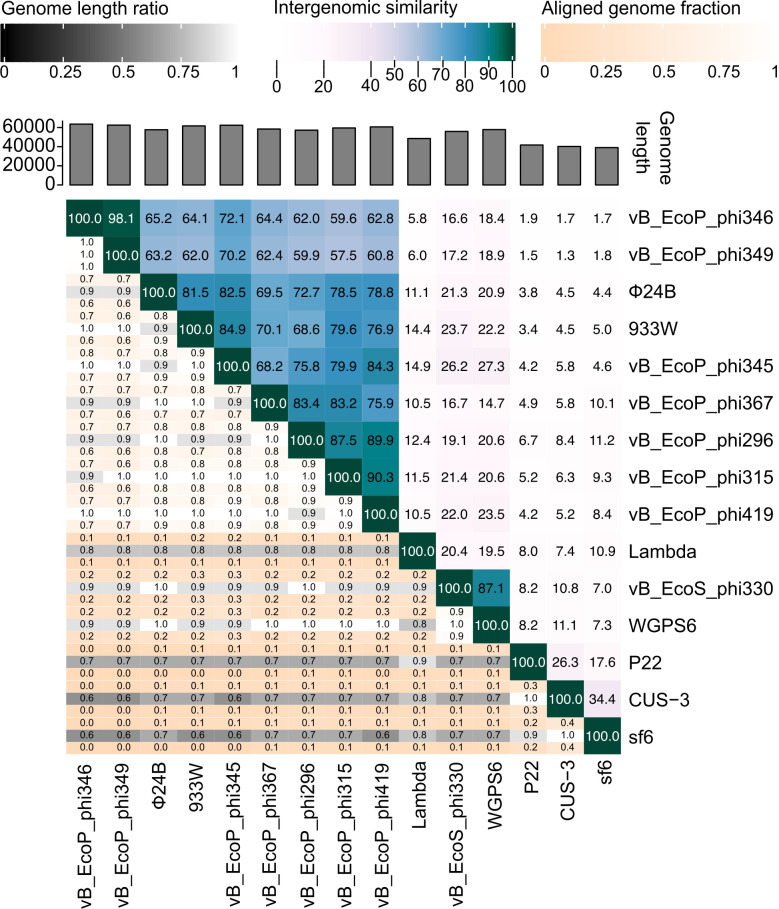
VIRIDIC heatmap of pairwise intergenomic similarities between the phages from this study compared to podoviruses 933W and Ф24_B_, siphoviruses Lambda, and WGPS6 and P22-like phages P22, CUS-3, and sf6. On the right side, the intergenomic similarity values are indicated in percentage with a blue color gradient (the darker the more similarity). On the left side, the alignment indicators are shown with three different values, from top to bottom: the aligned genome fraction for the genome in that row—genome length ratio—the aligned genome fraction for the genome in that column.

**Fig 3:**
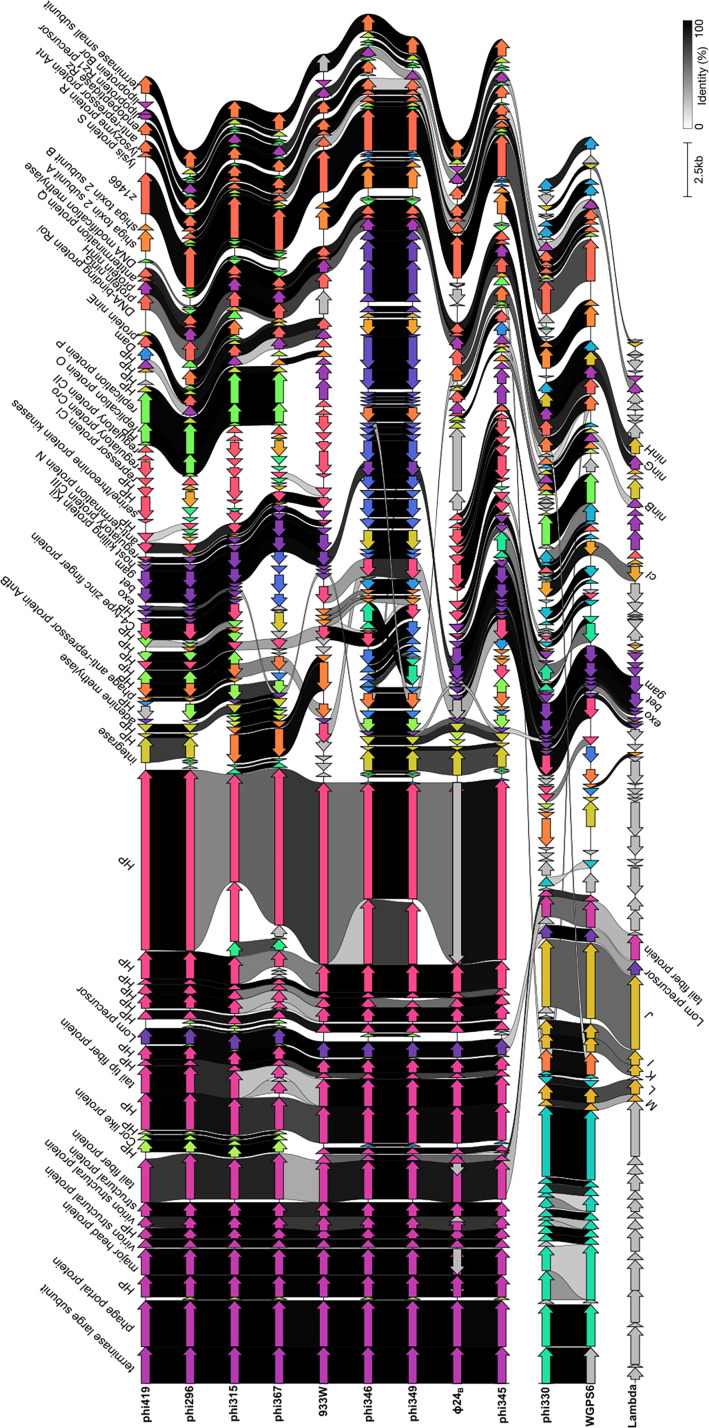
Linear comparison of the genome organization of the eight coliphages in this study compared with bacteriophages 933W, Ф24B, WGPS6, and Lambda based on amino acid sequence. The genomes were aligned using Clinker. Genes or gene groups are grouped into the same cluster based on sequence similarity and are colored identically. CDSs are linked with black bars according to the percentage of amino acid identity. HP: hypothetical protein.

To explore and quantify intergenomic similarities between the phages, a heatmap was generated using VIRIDIC that illustrates the degrees of similarity across the newly isolated phages of this study and already known, well-characterized phages. (Fig. 2). The additional phages used for genome comparison are podophages 933W (AF125520) and ϕ24_B_::Cat (HM208303), and siphophages Lambda (J02459) and Pankowvirus WGPS6 (NC_049945). The latter was selected because it shares the most sequence identity with phi330. The same phage genomes were also linearly compared based on the translated amino acid sequences of their coding sequences using Clinker (Fig. 3) (28) and circularly using BRIG (Fig. S1). Long-tailed phi330 is, as expected from its morphological features, most distinct from the other phages in this study. Phi330 nevertheless shares its *stx* operon with phi345, phi346, phi349, and phi419, which is absent in phi315, phi296, and phi367 (Fig. 3). All *stx*-encoding phages contain subtype *stx2a*. Among the late phage genes, a conserved open-reading frame was found in all phages. This gene is identical to the z1466 gene found in phage 933W and is homologous to a Neu5,9Ac_2_ esterase (NanS) encoding gene in *E. coli*. The Lambda Red recombination genes (*exo*, *bet,* and *gam*) are conserved across all phages (29), except for phi346 and phi349, where only the exonuclease gene is conserved compared with the other phages. Lambda genes *lom* and *bor* are also conserved across the different phages (except for *bor* in phi330) (30). The *lom* and *bor* genes are expressed during lysogeny and confer advantages to the lysogenic bacterial cells for survival in mammalian hosts, contributing to their pathogenicity.

In agreement with their similar morphologies, structural proteins, such as the major head protein, virion structural protein, and tail fiber proteins, are conserved among all podoviruses, yet differ from the siphoviruses. Mass spectrometry analysis was conducted on the CsCl-purified phages to confirm the presence of the structural proteins predicted by the ORFs in the different genome sequences. Mass spectrometry-derived protein maps for the major head protein, tail fiber protein, and virion structural protein for all podoviruses show a good peptide coverage over the full length of the proteins (Fig. S3). The podoviruses share a high degree of sequence identity (60%–85%) with the short-tailed Stx-converting phages 933W and ϕ24_B_, particularly in the genes encoding structural proteins. In contrast, the podoviruses analyzed in this study share only 20% sequence identity with phi330 and 6% (phi346) to 15% (phi345) identity with Lambda, whereas the long-tailed phi330 phage shows 20% sequence identity with Lambda. Some degree of sequence identity is observed between Lambda and phi330, both siphoviruses, for the tail proteins (tail components M, L, K, I, and J in Lambda).

In phages phi346 and phi349, two orphan type II antitoxins belonging to the ParD and HigA families were identified, which are absent in the other phages. However, it is unclear if these antitoxins are part of phage anti-defense mechanisms. The phage genomes were subjected to DefenseFinder to search for anti-defense systems (31), but these antitoxins are not recognized as anti-defense systems. DefenseFinder detected anti-restriction-modification systems in all phage genomes except phi330, protecting the phage DNA from host restriction enzymes via DNA methyltransferase (MTase). Additionally, restriction alleviation (Ral) proteins are found in phi296 and phi367. Gam, which is present in all phages, is also considered an antidefense system by inhibiting host RecBCD nuclease, thereby preventing degradation of linear phage DNA (32).

Interestingly, both integrase and the immunity region encoding *cI*, *cro,* and *cII* show specific clustering patterns, independent of phage morphology, as can be visualized in Fig. S1. For integrases, three different clusters were observed. Cluster 1 contains phages phi296, phi345, phi346, phi349, and phi419; cluster 2, consisting of phi315 and phi367, shares another integrase variant, both distinct from phi330, which carries a third variant. This suggests that the prophages integrate at different sites in the bacterial genome. Compared with the known phages, cluster 1 shares around 85% and 35% sequence identity with ϕ24_B_ and WGPS6, respectively, whereas the integrase gene of Cluster 2 is conserved in phage 933W and shares 60% with phi330, all completely different from the integrase gene from Lambda, sharing no sequence identity. The immunity region, comprising the *cI* and *cro* regulatory genes, forms different subgroups: ϕ24_B_, 933W, phi315, phi345, and phi419 share one variant; phi346 and phi349 another; phi296 and phi367 a third, and phi330 and WGPS6 a fourth variant, sharing 26.7% sequence identity with Lambda, as *cI* is conserved here. The CII activator protein also follows this clustering but is additionally conserved in phi330, WGPS6, ϕ24_B_, 933W, phi315, phi345, and phi419. These different clustering patterns display a modular structure, highlighting the typical mosaic pattern that characterizes lambdoid phages.

### Identification of the phage host receptors

**Fig 4:**
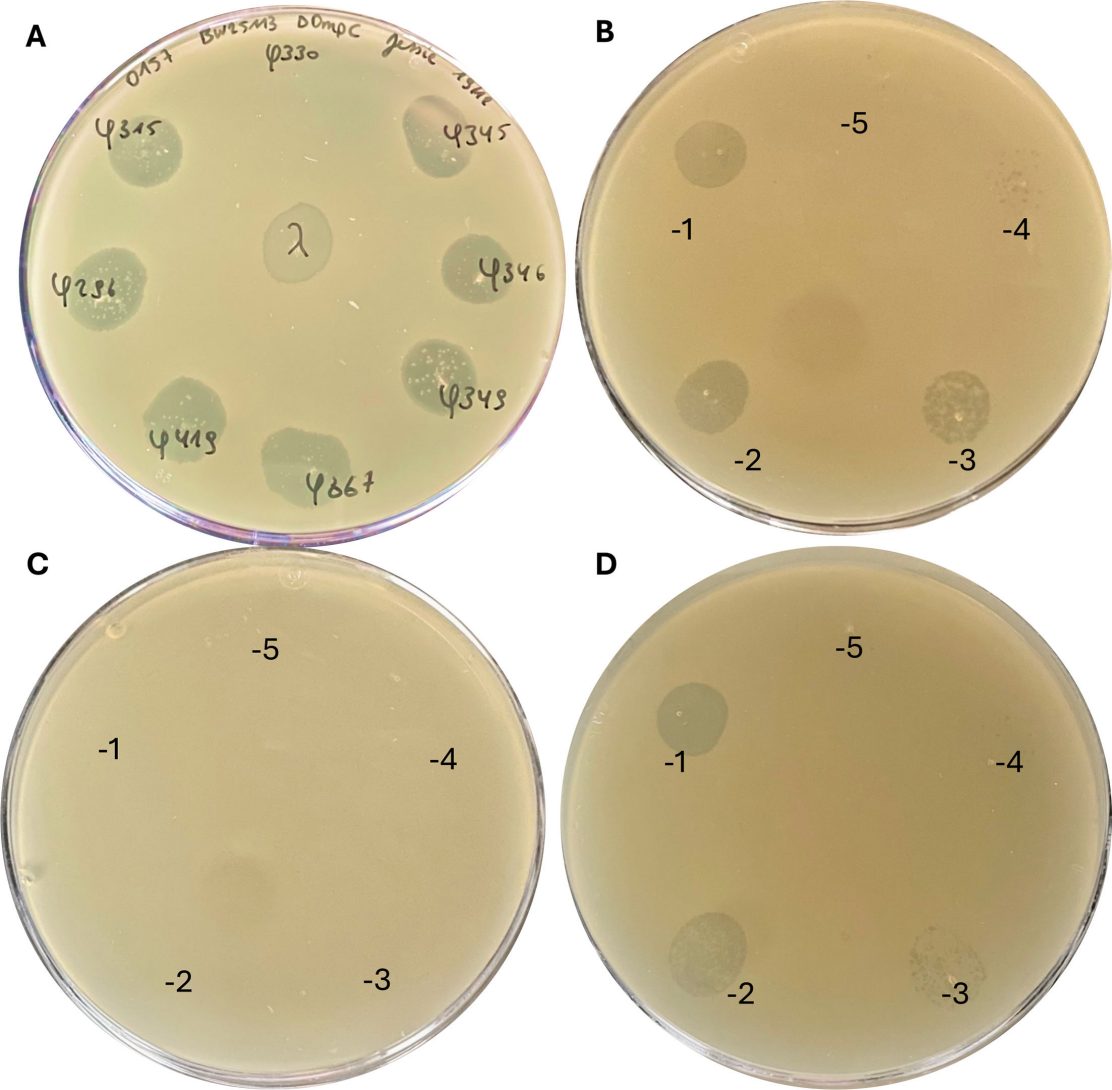
Plaque assays of phages on *E. coli* BW25113 ΔompC768::kan. (**A**) Plaque assay of all phages from this study and lambda on BW25113 ΔompC768::kan. (**B**) Plaque assay of phi330 on *E. coli* BW25113. (**C**) Plaque assay of phi330 on *E. coli* BW25113 ΔompC768::kan. (**D**) Plaque assay of phi330 on a plate supplemented with IPTG and *E. coli* BW25113 ΔompC768::kan + Plac ompC.

To determine the receptors for the phages, spot assays of the different phages were performed using 54 *E. coli* BW25113 Keio mutants (33), each with a knockout of an outer membrane protein, as listed in Table S2. All phages produce lysis zones on all strains, with the exception of phi330, which shows no lysis on *E. coli* BW25113 Δ*ompC768::kan* mutant (Fig. 4A). This suggests that phi330 targets the outer membrane porin C (OmpC). To confirm that OmpC is the receptor of phi330, *ompC* was re-inserted into the chromosome of the *ompC*-deleted *E. coli* strain under control of the lac promoter (P*lac-ompC*) via Tn7 transposition (20). Plaque assays were consequently done for phi330 on *E. coli* BW25113 (Fig. 4B), BW25113 Δ*ompC768::kan* (Fig. 4C), and BW25113 Δ*ompC768::kan +* P*lac* ompC (Fig. 4D). Plaques were observed on both BW25113 and BW25113 Δ*ompC768::kan* complemented with P*lac-ompC*, but not on BW25113 Δ*ompC768::kan*, proving that phi330 indeed targets OmpC as its receptor.

AlphaFold3 prediction of the putative tail fiber protein of phi330 shows it to be structurally similar to the central tail fiber protein of bacteriophage 434 targeting OmpC and the J protein of bacteriophage Lambda targeting maltoporin LamB (Fig. S4) (34–36). Analogously to lambda and bacteriophage 434, the tail fiber protein of phi330 forms a trimer to dock to the trimeric OmpC porin. The receptor binding domains of the three proteins share 35%–40% pairwise sequence identity and indicate a common ancestor despite the difference in phage receptors. One hairpin of phi330 (E953-S962) points into the OmpC barrel, whereas a second hairpin (A1015–P1020) makes extensive contacts with the rim of the OmpC barrel. For bacteriophage 434, a third loop is also predicted to make contact with OmpC. However, the corresponding loop in phi330 (T918–Y921) is significantly shorter than the bacteriophage 434 loop and is not predicted to make contacts with OmpC. Both sequence and conformation for the corresponding hairpin loops are not conserved between the 434 and phi330 proteins, and the details of the interactions differ (Fig. S4D and E).

**Fig 5:**
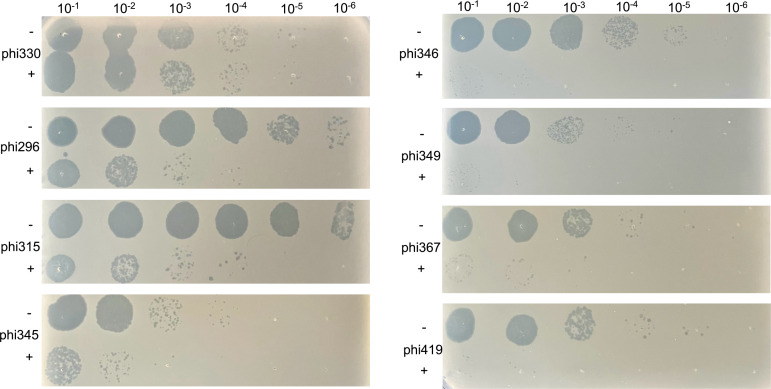
Plaque assay of all phages on *E. coli* K514 with (+) and without (−) BamA preincubation. Serial dilutions of BamA-treated bacteriophages (see the Material and methods) were spotted on *E. coli* K514 bacteria.

Given the observed differences in tail morphology and tail fiber genes, it is not surprising that phi330 targets a different receptor than the podoviruses studied here. Previous studies have shown that certain short-tailed Stx phages use the BamA (YaeT) protein as a receptor (37). As BamA is an essential protein, a knockout mutant is lethal to the cell. Therefore, a competition experiment was designed where an excess of purified BamA was pre-incubated with the different phages, followed by a plaque assay on *E. coli* K514 (Fig. 5; Fig. S5 and Table S3). Different replicates of this experiment show variation in the titer of the phages, which is not unusual for EHEC phages (38). Nevertheless, a significant reduction in infection capacity of two to three orders of magnitude is observed in most experiments for all podoviruses when pre-incubated with BamA compared with when BamA is absent. In contrast, no reduction is observed for phi330, which was included as a negative control. These findings strongly suggest that the seven podoviruses all target BamA as their receptor. This is further supported by the sequence identity of the tail fiber protein, which is shared among the seven podoviruses and short-tailed phages, such as 933W and ϕ24_B_, both shown previously to target the BamA protein (10, 37).

## DISCUSSION

Lambdoid phages, including EHEC phages, are tailed dsDNA phages that share a similar core genetic organization with the archetypic phage Lambda (9). When comparing the eight phages from this study to Lambda, the long-tailed phi330 shows the most, although still limited similarity to the Lambda genome (around 20% nucleotide sequence identity). Genomic heterogeneity within temperate EHEC phages is common and arises from various recombination events occurring between lambdoid phages, generating new hybrid phages with mosaic patterns. This dynamic recombination contributes significantly to the evolution and adaptability of these phages, creating a diverse pool of phages that significantly impact the ecology and virulence of bacterial populations. Lambdoid phage genome sizes range from 28.7 to 71.9 kb, with typical sizes of around 40 to 60 kb (39). This is consistent with the sizes of the phages studied here.

The unique sequence and assembly obtained for phages phi330, phi345, phi346, phi349, and phi419 suggest that these phages are likely correctly sequenced and assembled. Still, EHEC strains can contain multiple phages that can form hybrids, and unfortunately, we do not have sequence data available from the host genome. For the non-Stx phages phi296, phi315, and phi367, we initially observed issues regarding possible phage purity after nanopore sequencing, raising the possibility that they represented a mixture of closely related phages. Furthermore, there is a non-zero risk that some of the phages sequenced could be assembled as chimeras. Indeed, poly-lysogeny is a common phenomenon, and multiple phages might be (spontaneously) induced from their lysogens. To solve the issue of different contigs that led to different possible assemblies, primers were designed to be located at the border in the different contigs, and PCR was performed on single plaques. The resulting PCR fragments were subsequently Sanger-sequenced. This led to a final assembly for each phage that was further characterized and used for comparison studies.

Although we are quite confident that our sequences correspond to unique phages, it cannot be concluded with absolute certainty that each phage is truly unique. Especially when working with phages that naturally form hybrids and where it might be difficult to distinguish from chimeras, as is the case with lambdoid phages. Working with such phages needs to be split in time and space to avoid cross-contamination (40). Improper phage isolation and purification can lead to a mixture of subclones in a phage sample.

To study our phages in a broader *E. coli* context, their genomes were also compared using VIRIDIC to P22-like phages (Fig. 2): *Salmonella* phage P22, *Shigella* phage Sf6, and *E. coli* phage CUS-3 (accession numbers BK000583.1, AF547987.1, and CP000711.1, respectively) (41). These P22-like phages are each representative of distinct groups of P22-like phages based on their coat proteins (42). Although both P22 and Lambda are known to form hybrids with multiple phages and have a similar genomic organization and regulation, they are still significantly different in genetic content (43). Sequence identity of the phages in this study compared with P22-like phages is limited and generally between 1.5% and 11%. The lowest values are obtained for phi346 and phi349, which share the least similarity with P22-like phages. Phage Sf6 shares most sequence identity with podophage phi296 (around 11%) and includes genes *cII*, *O* and *P*, *ninB,* and *ral*. Phage CUS-3 shares most similarity with siphophage phi330 (also around 11%), mainly restricted to the *O* and *P* genes. Although limited, the observed sequence identity may theoretically be sufficient to permit homologous recombination between these phages, provided they infect the same bacterial host, as only 50 base pairs of homology can facilitate recombination under certain conditions. However, they would often not result in viable progeny as they are functionally incompatible.

Morphologically, variation also exists within Stx phages. The most predominant head feature of Stx phages is the icosahedral shape, although elongated heads have also been documented (44). Tail structures vary between long contractile, long non-contractile, or short tails, contributing further to the Stx phage diversity, reflecting their classification primarily within the morphological groups of podo- and siphoviruses, whereas only a few representatives have been classified as myoviruses (10, 37, 45). Therefore, we expected to classify our phages into either of these two morphological groups, which is confirmed by the negative stain TEM images.

Tail fiber proteins play a crucial role in host specificity and adsorption. The majority of Stx podoviruses such as 933W and ϕ24_B_ utilize highly conserved tail fiber proteins for host adsorption (37, 46), which is also conserved within the short-tailed phages studied here. The highly conserved and essential BamA (YaeT) protein frequently serves as a receptor for short-tailed Stx phages. It was shown that more than 70% of Stx phages share a gene linked to short-tailed morphology responsible for binding the BamA protein (37). For the short-tailed phages in our collection, it was demonstrated via a competition assay that BamA is indeed their receptor. As BamA is critical for bacterial cell viability, its presence on bacterial host cells is ensured, and mutations or downregulation of the *bamA* gene are very unlikely. The stability of BamA ensures the phage’s propagation, contributing to its evolution and spread within STEC. Evidently, long-tailed Stx phages recognize different receptors, including outer membrane proteins like OmpC (47). Phi330, characterized in this study, also targets the non-essential OmpC protein, as shown by the inability to infect an *ompC* knockout mutant and confirmed by the restorability of infection when complementation of *ompC* is established. Comprehending the specificity of these phage-host interactions offers insights into potential therapeutic strategies to mitigate phage-mediated horizontal gene transfer, reducing the host’s virulence by, for example, targeting phage receptors like BamA or OmpC, disrupting early stages of phage infection.

Regarding the toxin production, not all EHEC prophages encode the Shiga toxin. As polylysogeny is very common in EHEC strains, it is likely to find both Stx and non-Stx prophages in EHEC’s genome (48). Still, their genetic organization remains highly similar (49). This study also demonstrates this pattern, as the *stx* operon is present in most, but not all, isolated EHEC phages; however, their genetic organization is identical to that of the Stx-converting phages. Different Stx types exist, namely Stx1 and Stx2, each composed of several subtypes. Although Stx1 is more homogeneous and only consists of three subtypes (Stx1a, Stx1c, and Stx1d), Stx2 is more heterogeneous, consisting of subtypes Stx2a, Stx2b, Stx2c, Stx2d, Stx2e, Stx2f, and Stx2g. Additional subtypes have recently been described for Stx2–Stx2h to Stx2k (50, 51). There is also a difference in virulence between the two Stx types. Stx2 is typically associated with more severe cases of disease, including HUS, due to its higher potency and more efficient induction, as observed with subtypes like Stx2a (52). In our collection, *stx*-encoding phages contain the *stx2a* subtype. STEC or EHEC expressing different subtypes of Stx2 are often associated with different animal reservoirs (53). Besides *stx*, other prophage genes have been described that might contribute to EHEC pathogenicity (54). Another gene that is often present in Stx and non-Stx phages of pathogenic EHEC is open reading frame z1466, which was also found in all our isolated phages. This gene is homologous to *yjhS* (*nanS*) gene in *E. coli,* encoding a 9-O-acetyl-N-acetylneuraminic acid (Neu5,9Ac_2_) esterase (55). Neu5,9Ac_2_ esterase facilitates the growth sialic acid found in mammalian mucosal tissues. This prophage-encoded gene confers an ecological benefit to the lysogen to grow within the human host (56). Furthermore, in two very similar phages (phi346 and phi349), two genes encoding orphan type II antitoxins belonging to the ParD and HigA families were identified. The biological relevance of this prophage-encoded antitoxin is unclear. Possibly, they serve to counteract a corresponding host-encoded toxin that is activated upon phage infection. Several cases are known where phages encode an alternative antitoxin that mimics the role of the native antitoxin that is degraded upon phage infection (57–60). There are, however, no verified examples known of classic antitoxins (that pair with a specific cognate toxin in a TA pair) that take on such a role. Screening phage genomes in DefenseFinder for anti-defense mechanisms exposed mainly anti-RM and anti-RecBCD systems, which is expected as anti-RM is the most abundant anti-defense mechanism in phages (61). Anti-CRISPR (Acr) proteins were not found in our phage genomes via DefenseFinder.

In conclusion, the complexity of EHEC phages’ genomic content, morphological diversity, and host interactions underscores their evolutionary adaptability and the contributions of these prophages to bacterial virulence. Advances in understanding phage-host interactions and prophage-encoded auxiliary genes present opportunities for the development of innovative therapeutic strategies.

## Data Availability

Phage genomes have been deposited in GenBank with the accession numbers in Table 2. The mass spectrometry proteomics data have been deposited to the ProteomeXchange Consortium via the PRIDE (62) partner repository with the data set identifier PXD061229 with doi 10.6019/PXD061229.

## SUPPLEMENTAL MATERIAL

Data S1 spectrum.00597-25-s0001.bam Sequence alignment map of phi296 containing the nanopore assembly aligned with the different contigs and Sanger sequences connecting these contains.

Data S2 spectrum.00597-25-s0002.bam Sequence alignment map of phi315 containing the nanopore assembly aligned with the different contigs and Sanger sequences connecting these contains.

Data S3 spectrum.00597-25-s0003.bam Sequence alignment map of phi367 containing the nanopore assembly aligned with the different contigs and Sanger sequences connecting these contains.

Supplemental material spectrum.00597-25-s0004.docx Fig. S1 to S5; Tables S1 to S3.

ASM does not own the copyrights to Supplemental Material that may be linked to, or accessed through, an article. The authors have granted ASM a non-exclusive, world-wide license to publish the Supplemental Material files. Please contact the corresponding author directly for reuse.
