# Supplementary material for: Isolation and characterization of bacteriophages from clinical enterohemorrhagic Escherichia coli strains
Source: Microbiol Spectr. 2025 Sep 5;13(10):e00597-25. doi: 10.1128/spectrum.00597-25 (PMC12502783; doi:10.1128/spectrum.00597-25)
Supplement: Supplemental material — Fig. S1 to S5; Tables S1 to S3. [file spectrum.00597-25-s0004.docx]

***Figure S1:*** VIRIDIC heatmap of pairwise intergenomic similarities between the phages from this study compared to *bacteriophages 933W, Ф24B, WGPS6 and Lambda*. **A**: Comparison of the integrase gene. **B**: Comparison of the immunity region containing cI, cro and cII. On the right side the intergenomic similarity values are indicated in percentage with a blue color gradient (the darker the more similarity). On the left side the alignment indicators are shown with three different values, from top to bottom: the aligned genome fraction for the genome in that row – genome length ratio – the aligned genome fraction for the genome in that column.


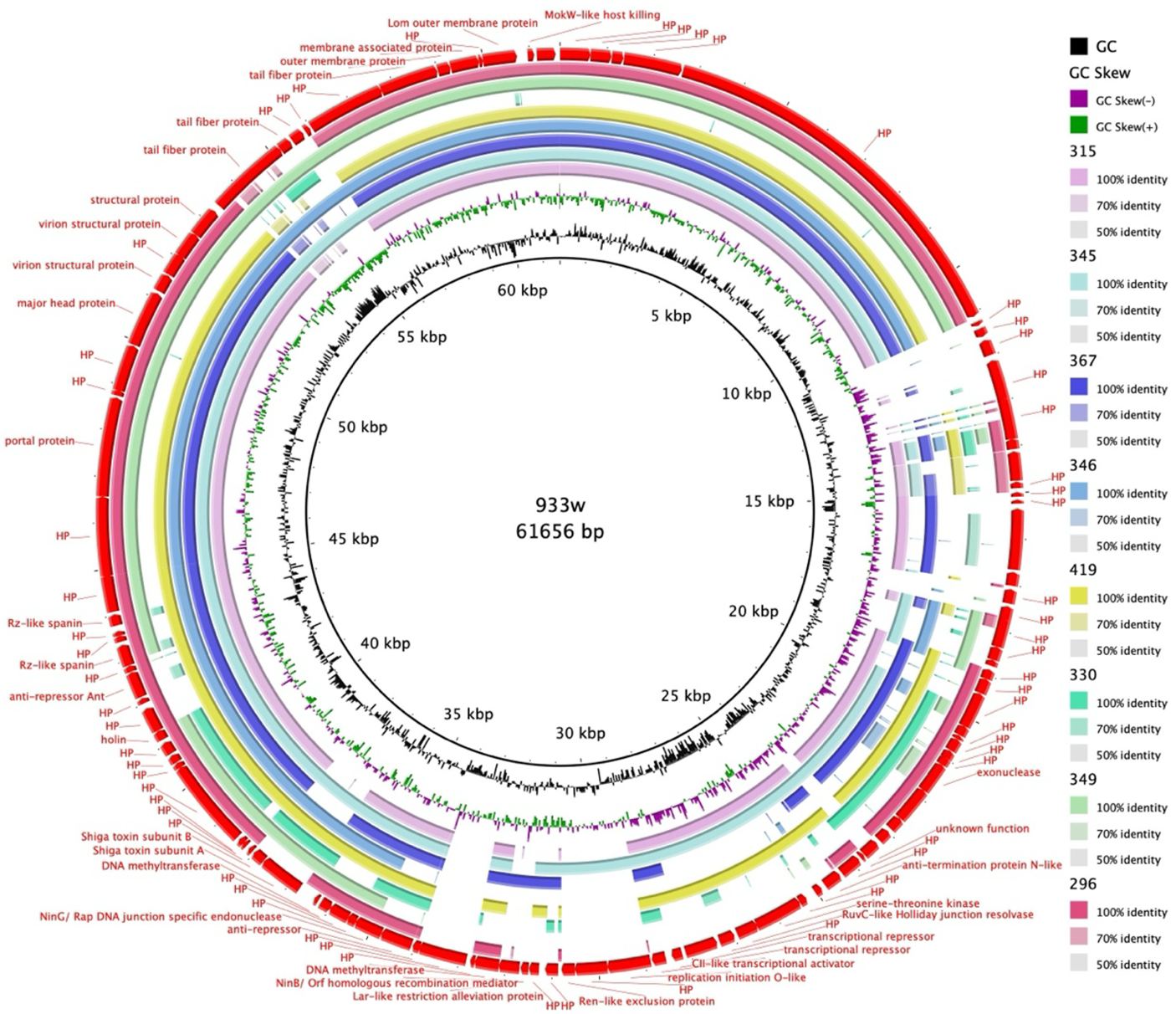


***Figure S2****: BLAST Ring Image Generator (BRIG) comparison of the eight phages compared to the reference genome of bacteriophage 933W, based on nucleotide sequences. HP: hypothetical protein.*

******

***Figure S3:*** *Protein maps with peptide coverage from mass spectrometry experiments for all short-tailed phages in this study.*


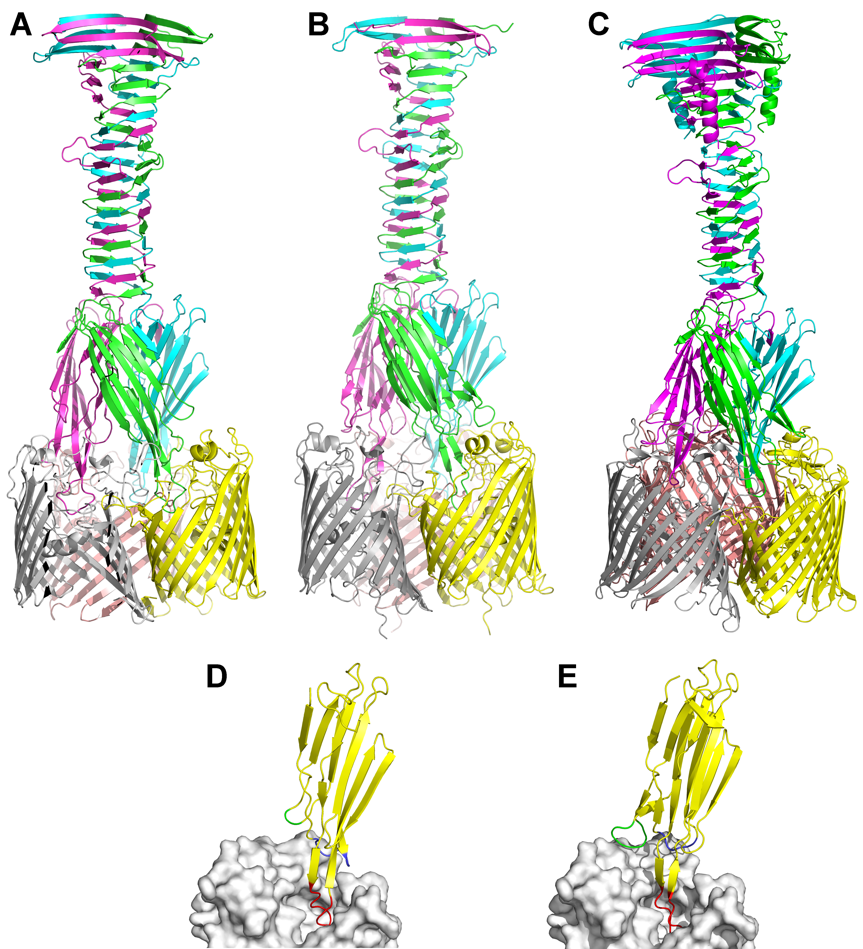


***Figure S4****. AlphaFold3 prediction of receptor recognition of phi330.* ***A****: Cartoon representation of the AlphaFold3 prediction of the putative tail fiber protein trimer of phi330 (residues T765-S1042) bound to the OmpC trimer. Subunits of the tail fiber protein are shown in cyan, green and magenta. OmpC subunits are colored yellow, grey and salmon.* ***B.*** *Similar view of the corresponding AlphaFold3 prediction of the complex between the tail fiber protein of bacteriophage 434 (residues G872-R1136) and OmpC. Colors are as in panel A.* ***C.*** *Similar representation of the structure of the bacteriophage lambda J protein (residues D810-V1132) bound to its receptor LamB. Colors are as in panel A.* ***D.*** *Close-up view of the receptor binding domain of one subunit of the phi330 tail fiber protein interacting with OmpC. The hairpin loop E953-S962 that points inwards in the OmpC barrel is shown in red. The hairpin loop touching the rim of the OmpC barrel (A1015-P1020) is shown in blue, while the third loop that is relevant for phage 434 and lambda but is not predicted for phi330 to interact with OmpC is colored green.* ***E.*** *Similar view for the corresponding complex of bacteriophage 343 and an identical coloring scheme for the loops that interact with OmpC. The differences in conformation of the interacting hairpin loops for the receptor domains from phi330 and 434 can be clearly distinguished.*

***Figure S5****: Plaque assay of triplicates of all phages on E. coli K514 with (+) and without (-) BamA preincubation.*

***Table S1****: List of clinical isolates of enterohaemorrhagic E. coli (EHEC) from which phages were isolated and their corresponding titers. The phages indicated in bold are the phages that are further characterized in this study.*

| **Clinical isolate** | **Phage** | **Titer (PFU/mL)** |
| --- | --- | --- |
| EH 118 |  |  |
| EH 345 | **phi345** | 4 × 10^10^ |
| EH 346 | **phi346** | 4 ×10^11^ |
| EH 349 | **phi349** | 1.5 × 10^11^ |
| EH10 |  |  |
| EH12 |  |  |
| H19 | phi19 | No single plaques |
| H30 | phi30 | No single plaques |
| E57 |  |  |
| 7279 |  |  |
| EH 296 | **phi296** | 10^10^ |
| EH 315 | **phi315** | 10^10^ |
| EH 337 |  |  |
| EH 367 | **phi367** | 1.8 × 10^11^ |
| EH 406 |  |  |
| EH 419 | **phi419** | 1.5 × 10^11^ |
| EH 422 |  |  |
| EH 449 |  |  |
| EH 478 |  |  |
| EH 502 |  |  |
| EH 505 | phi505 | Titer too low |
| E32511 |  |  |
| EH 517 |  |  |
| EH 521 |  |  |
| EH 525 | phi525 | No single plaques |
| EH 526 |  |  |
| EH 327 |  |  |
| EH 255 |  |  |
| EH 330 | **phi330** | 10^9^ |

***Table S2****: List of E. coli BW25113 Keio mutants used for receptor identification of the different phages.*

| *lamB732* | *fepA721* | *nfrA777* | *uidC763* | *ybfM729* | *ydiY721* |
| --- | --- | --- | --- | --- | --- |
| *ompC768* | *fhuA766* | *nfrB778* | *yfaZ731* | *yhdV785* | *ydbJ742* |
| *ompA772* | *btuB754* | *ompL737* | *rcsF721* | *yjbF723* | *yoaF789* |
| *ompF746* | *fadL752* | *ompN740* | *csgG778* | *mipA779* | *yfiB768* |
| *ompG756* | *fecA758* | *ompX786* | *pldA766* | *yiaT768* | *cusC781* |
| *ompT774* | *fhuE764* | *ompW764* | *nlpC782* | *flu-768* | *amn-756* |
| *lpp-752* | *tonB760* | *fiu777* | *ybeQ777* | *yaiT738* | *yehL744* |
| *phoE759* | *tolC732* | *ygiB733* | *nlpD747* | *yddL765* | *yceK721* |
| *tsx-773* | *tonB760* | *yfeN738* | *yfgH754* | *fimD785* | *yidQ724* |

***Table S3****:* Quantitative decrease of phage infectivity for the BamA competition assay based on the efficiency of plaquing (EOP) for different replicates of podophages compared to phi330 as negative control. Titers with and without BamA pre-incubation are mentioned in PFU/mL and their ratio is calculated.

| **phi330** | **Replicate 1** | **Replicate 2** | **Replicate 3** | **Replicate 4** |
| --- | --- | --- | --- | --- |
|  | Phagestock 1 | Phagestock 2 | Phagestock 2 | Phagestock 2 |
| **- BamA** | 8 × 10^8^ | 1,1× 10^8^ | 4 × 10^7^ | 3 × 10^6^ |
| **+ BamA** | 2 × 10^9^ | 1 × 10^8^ | 3 × 10^7^ | 3 × 10^6^ |
| **Ratio** | 0,4 | 1,1 | 1,3 | 1 |
| **phi296** | **Replicate 1** | **Replicate 2** | **Replicate 3** | **Replicate 4** |
|  | Phagestock 1 | Phagestock 2 | Phagestock 2 | Phagestock 2 |
| **- BamA** | 1 × 10^9^ | 8 × 10^9^ | 8 × 10^9^ | 1,2 × 10^9^ |
| **+ BamA** | 4 × 10^5^ | 1,4 × 10^7^ | 7 × 10^6^ | 2 × 10^6^ |
| **Ratio** | 2,5 × 10^3^ | 3,6 × 10^2^ | 1,1 × 10^3^ | 6 × 10^2^ |
| **phi315** | **Replicate 1** | **Replicate 2** | **Replicate 3** | **Replicate 4** |
|  | Phagestock 1 | Phagestock 2 | Phagestock 2 | Phagestock 2 |
| **- BamA** | 9,6 × 10^9^ | 7 × 10^9^ | 8 × 10^9^ | 1,2 × 10^9^ |
| **+ BamA** | 3,2 × 10^6^ | 1,6 × 10^7^ | 1,1 × 10^7^ | 2 × 10^6^ |
| **Ratio** | 3 × 10^3^ | 4,4 × 10^2^ | 7,3 × 10^2^ | 6 × 10^2^ |
| **phi345** | **Replicate 1** | **Replicate 2** | **Replicate 3** | **Replicate 4** |
|  | Phagestock 1 | Phagestock 2 | Phagestock 2 | Phagestock 2 |
| **- BamA** | 6 × 10^8^ | 9 × 10^7^ | 4 × 10^7^ | 2,2 × 10^7^ |
| **+ BamA** | 1,6 × 10^5^ | 1,8 × 10^5^ | 9 × 10^5^ | 2 × 10^5^ |
| **Ratio** | 3,7 × 10^3^ | 5 × 10^2^ | 4,4 × 10^1^ | 1,1 × 10^2^ |
| **phi346** | **Replicate 1** | **Replicate 2** | **Replicate 3** | **Replicate 4** |
|  | Phagestock 1 | Phagestock 2 | Phagestock 2 | Phagestock 2 |
| **- BamA** | 1 × 10^8^ | 6 × 10^8^ | 3 × 10^9^ | 1.8 × 10^9^ |
| **+ BamA** | 4 × 10^5^ | 4 × 10^5^ | 2 × 10^5^ | 2 × 10^5^ |
| **Ratio** | 2.5 × 10^2^ | 1.5 × 10^3^ | 1.5 × 10^4^ | 9 × 10^3^ |
| **phi349** | **Replicate 1** | **Replicate 2** | **Replicate 3** | **Replicate 4** |
|  | Phagestock 1 | Phagestock 2 | Phagestock 2 | Phagestock 2 |
| **- BamA** | 1.5 × 10^6^ | 4 × 10^7^ | 8 × 10^7^ | 1.6 × 10^8^ |
| **+ BamA** | < 1 × 10^3^ | 5 × 10^4^ | 8 × 10^4^ | 9 × 10^4^ |
| **Ratio** | ≥1.5 × 10^3^ | 8 × 10^2^ | 1 × 10^3^ | 1.8 × 10^3^ |
| **phi367** | **Replicate 1** | **Replicate 2** | **Replicate 3** | **Replicate 4** |
|  | Phagestock 1 | Phagestock 2 | Phagestock 2 | Phagestock 2 |
| **- BamA** | 3 × 10^9^ | 4 × 10^7^ | 2 × 10^6^ | 2.8 × 10^7^ |
| **+ BamA** | 3 × 10^6^ | 6 × 10^5^ | 5 × 10^4^ | 1.2 × 10^4^ |
| **Ratio** | 1 × 10^3^ | 6,7 × 10^1^ | 4 × 10^1^ | 2.3 × 10^3^ |
| **Phi419** | **Replicate 1** | **Replicate 2** | **Replicate 3** | **Replicate 4** |
|  | Phagestock 1 | Phagestock 2 | Phagestock 2 | Phagestock 2 |
| **- BamA** | 3.3 × 10^6^ | 6 × 10^7^ | 3 × 10^7^ | 1.3 × 10^7^ |
| **+ BamA** | < 1 × 10^3^ | 4 × 10^4^ | 9 × 10^4^ | 6 × 10^3^ |
| **Ratio** | ≥3.3 × 10^3^ | 1.5 × 10^3^ | 3.3 × 10^2^ | 2.2 × 10^3^ |
